# Technological Applications Contributing to Relieve Care Burden or to Sleep of Caregivers and People With Dementia: A Scoping Review From the Perspective of Social Isolation

**DOI:** 10.3389/fpubh.2022.797176

**Published:** 2022-03-29

**Authors:** Chantal Huisman, Emelieke Huisman, Helianthe Kort

**Affiliations:** ^1^Research Group Technology for Healthcare Innovations, Research Center Healthy and Sustainable Living, University of Applied Science Utrecht, Utrecht, Netherlands; ^2^Building Healthy Environments for Future Users Group, Department of the Built Environment, Eindhoven University of Technology, Eindhoven, Netherlands

**Keywords:** informal caregiver, dementia, technology, loneliness, sleep, social isolation

## Abstract

The need for care will increase in the coming years. Most people with a disability or old age receive support from an informal caregiver. Caring for a person with dementia can be difficult because of the BPSD (Behavioral and Psychological Symptoms of Dementia). BPSD, including sleep disturbance, is an important factor for a higher care load. In this scoping review, we aim to investigate whether technology is available to support the informal caregiver, to lower the care burden, improve sleep quality, and therefore influence the reduction of social isolation of informal caregivers of people with dementia. A scoping review is performed following the methodological framework by Arksey and O'Mally and Rumrill et al., the scoping review includes scientific and other sources (unpublished literature, websites, reports, etc.). The findings of the scoping review shows that there are technology applications available to support the informal caregiver of a person with dementia. The technology applications mostly contribute to lower the care burden and/or improve sleep quality and therefore may contribute to reduce social isolation. The technology applications found target either the person with dementia, the informal caregiver, or both.

## Introduction

In 2019 there were 703 million older persons aged 65 or over and in the coming decades, this number will double to more than 1.5 billion persons in 2050 (1) that, according to Alzheimer's Europe (2019), there are almost 9.8 million people with dementia in Europe (2). This number will almost double to 18.8 million people in 2050. With these numbers of older people (with dementia) the need for care will also increase. Most people with a disability or older age-dependent on support from an informal caregiver (a relative or friend) (3). In Europe, estimates suggest that approximately 80% of all long-term care is provided by informal caregivers (4). This also applies in the Netherlands (5).

Informal caregivers can experience a higher level of stress and depression. They experience a lower level of subjective wellbeing compared to non-caregivers and they encounter a greater risk of developing physical health problems. Also, they may experience a lack of social activities (6). Depending on the quality and duration of the relationship between the caregiver and the person with dementia, the experience of adverse physical and psychological health consequences may vary. With increasing numbers of years of care, the risk of physical and psychological health threats increases (7). Caring for a person with dementia can be difficult because of the behavioral and psychological symptoms of dementia (BPSD). BPSD is an important factor for a high care load of informal caregivers (8). BPSD affects about 90% of people with dementia at any given moment (9). BPSD comprises sleep disturbances, aggression, anxiety, and wandering (9). A review of Cross et al. (7) shows that informal caregivers perceive their situation as permanence and they experience a sense of being tied-in, being always alert, unappreciated, feeling trapped, like a prison, pulled in all directions, and at times, being in an unreal situation. These feelings with emotions of distress, hopelessness, depression, tiredness, exhaustion, frustration, guilt, negative thoughts, loss of patience, and isolation. The feeling of care burden may result in a decrease in the own quality of life of the informal caregiver but may also harm the person with dementia. The care burden and the possible decrease of quality of life make social and professional support essential (10). In the Netherlands, 15% of the informal caregivers experience loneliness when they live with a person with dementia (11).

This scoping review will focus on the sleep of informal caregivers of people with dementia, as a study indicates that they had poorer perceived sleep quality and shorter sleep duration than age-matched non-caregivers and population-based estimates (12). 50–70% of the informal caregivers of a person with dementia have sleep complaints (13). Several factors may disturb sleep, including environmental factors, physical and mental disorders (14). Sleep disturbance can worsen mental, physical, and cognitive health (12). In dementia the caregiver's sleep can be disturbed because of the stress and the increased cognitive burden, having to think and remember for two. Because of BPSD, people with dementia exhibit sleep disturbance and unhealthy sleep patterns, including short sleep duration, fragmented sleep, altered circadian rest/activity patterns, and an increase of sleep-disordered breathing (15). Research by Bubu et al. (15) shows that about 45% of persons with dementia have sleep disturbance. This can disturb the sleep of informal caregivers directly, and subsequently can worsen the ability to provide care effectively. In addition, decreased sleep quality has been associated with negative mindsets, depression, and anxiety which–in turn–can negatively affect the manner of care for the person with dementia (12).

**Figure 1:**
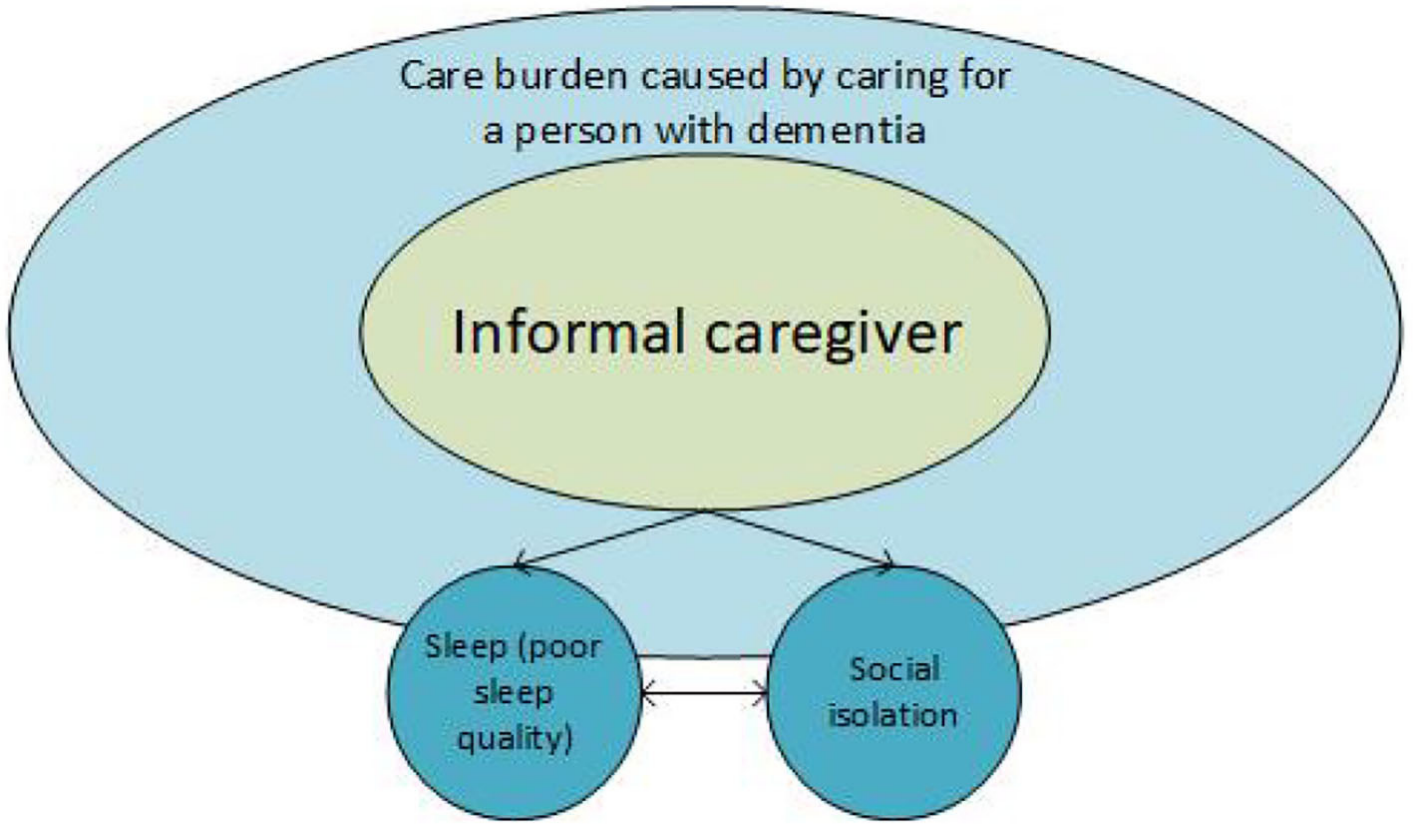
Simple illustration of factors; sleep quality; care burden and social isolation affecting informal caregivers of person with dementia.

The scoping review is based on the hypothesis that an informal caregiver is an important person in the life of a person with dementia. The care for a person with dementia who is still living at home can be tough, and even tougher when BPSD is involved; the latter being common. Being an informal caregiver often means experiencing a care burden, that can cause poorer sleep quality. In addition, the responsibility for taking care of a person with dementia in combination with poor sleep quality influences the social participation of the informal caregiver (Figure 1). The hypothesis is that (e)assistive technology should contribute to (a) lower the care burden, (b) improve sleep quality, and therefore (c) may positively influence the reduction of social isolation of informal caregivers of people with dementia.

The research question used for the scoping review is “Which technology is available to caregivers to reduce the negative effects of nightly activities from the person with dementia in the home setting?.”

## Methods

Following the methodological framework by Arksey and O'Mally (16) and Rumrill et al. (17), the scoping review includes scientific and other sources (unpublished literature, websites, reports, etc.). In short, the following steps need to be taken: (1) identify the initial research question(s), (2) identify the relevant studies, (3) study selection, (4) charting, and (5) collating, summarizing, and report the results and optional (6) consultation stage). In this first exploration, step 6 is not performed, because with this review an insight is gained about which technological innovations are studied and or on the market supporting the hypothesis.

### Identify and Selection

#### Search

A literature search (between January 2021 and May 2021) was conducted in Web of Science, the Association for Computing Machinery, the Institute of Electrical and Electronics Engineers (IEEE) Xplore Digital Library, ScienceDirect, and Cinahl. All libraries were searched for articles about dementia, technology, and sleep.

Depending on the database a search strategy was performed on abstract, title, and/or keywords. The following keywords were used: Dementia OR “Alzheimer's disease” AND Technology OR “internet-based intervention” OR innovation OR ICT OR robot AND Sleep OR “Sleep Initiation and Maintenance Disorders” OR “disorders of initiating and maintaining sleep” OR “Sleep disorder” OR “sleep disorders” OR Night OR “bedtime.” To adjust the search to the nature of the different databases, one change was necessary. In Science Direct the following keywords are used: Dementia OR “Alzheimer's Disease” AND Technology OR “internet-based intervention” OR innovation AND Sleep OR “Sleep disorder” OR Night, because of the restriction on Boolean operators. In addition to the database search, reference lists were reviewed to identify additional studies. Furthermore, reference lists of the relevant articles, social media, and other sources (e.g., reports from knowledge institutions and governments, overview pages) were searched for relevant publications and information utilizing. Google, Google Scholar, websites of knowledge institutions, YouTube, and Twitter are searched with terms like “technology,” “dementia,” “sleep.”

#### Selection

A first selection of the literature is made by reviewing only titles and abstracts. Including criteria are:

- Focus on dementia care.
- Focus on technology that can support the informal caregiver and/or the person with dementia before/during the night.
- Published in the English language.

The selection of studies was divided into two phases. In phase one, the first author (CH) preselected relevant studies based on title, abstract, and keyword; and in phase two, the co-authors (EH & HK) selected (the preselected) relevant articles based on abstract only.

Also, other sources were selected. Including criteria are based on language (English or Dutch), and relevance for the aim of this review. From these other sources, full-text versions were obtained and read in their entirety.

### Charting and Collecting

The literature studies and other sources included in this review are analyzed alongside the hypothesis that care for a person with dementia by informal caregivers is influenced by care burden, sleep quality, and therefore may cause social isolation. Besides that, the technology mentioned in the articles and other sources was categorized based on the so-called Pyramid of Technology, to indicate the level of the technology used (18). This view on technology describes the various levels at which technology may function in life in analogy with Maslow's Hierarchy of Needs, which describes human requirements (19). Similar to Maslow's model, technologies can move up and down. A lower stage needs to be fulfilled before a technology application can go to the next stage. New technologies are often seen as artificial but over time become accepted, familiar, and eventually even established. The different levels according to van Mensvoort are (1) envisioned (idea), (2) operational (tested small scale), (3) applied (available in practice), (4) accepted (daily life), (5) vital (second nature), (6) invisible (not even seen as technology), and (7) naturalized (human nature). The naturalized phase is rarely attained, most technology climb no higher than halfway up. When technology reaches this stage it either stabilizes or returns to lower levels because of new emerging technologies (18).

The first author analyzed and categorized the included studies based on the full papers. Articles were categorized by the authors, based on context or available outcomes on contributing to (a) lower the care burden, (b) improve sleep quality, and (c) may positively influence the reduction of social isolation of informal caregivers of people with dementia, using the four-eyes principle (20). In addition, the level of the technology or technologies mentioned was categorized as well based on the Pyramid of Technology. Furthermore, the type of study, number of participants, and duration of the study were noted. Both the second and third authors categorized the articles based on the title and abstract. No conflicts occurred between the scoring between the first and second author and between the first and third author.

Other sources are analyzed and categorized in the same way by the first author only.

## Results

**Figure 2:**
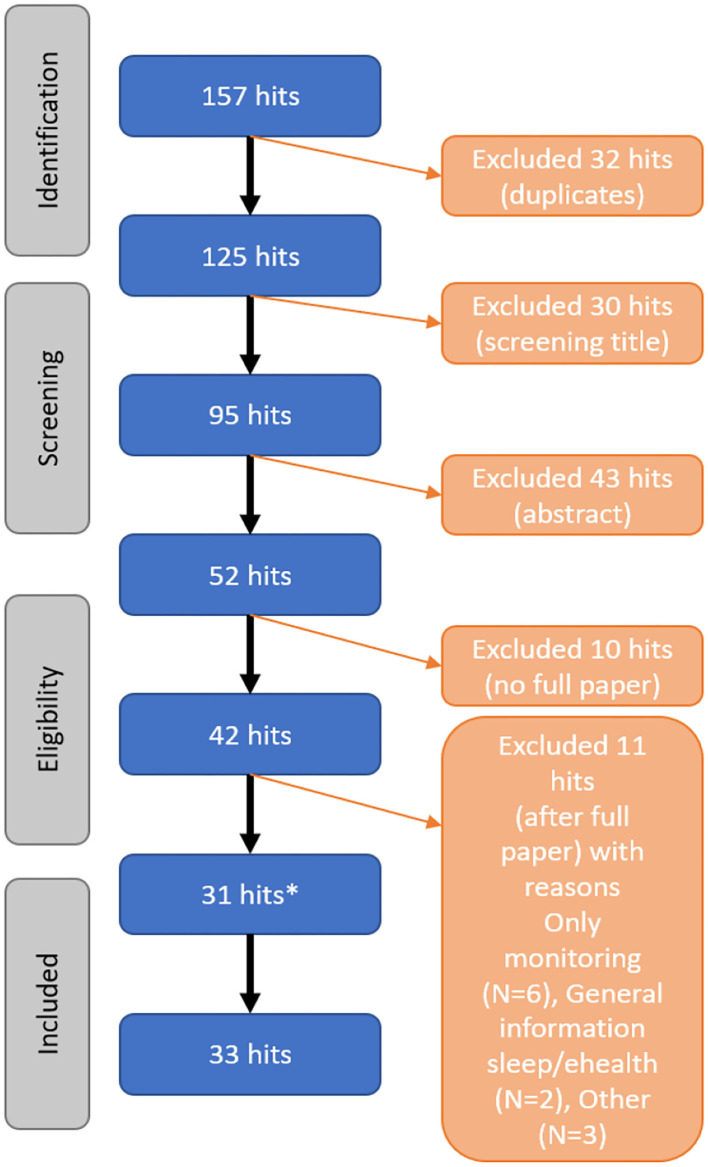
Flowchart of the screening process of the literature (*, added via Snowball-method).

A total of 157 articles are found, 32 of the articles were duplicates. Thirty articles are excluded based on title and 43 articles are excluded after reading the abstract. From 10 of the articles, no full paper was available. And after reading the full paper 11 articles are excluded. Via the snowball method, two additional articles are included. In total, 33 articles (scientific) are included in this scoping review, using the literature search described above (Figure 2).

Furthermore, and as described above, other sources were used to include available technology not yet described in the scientific literature resulting in the evaluation of 32 additional technology applications.

### Overall Results

Using the Pyramid of Technology, most (*N* = 26 out of 33) of the included studies described technology in the envisioned (1), operational (2), and/or the applied (3) phase. Only in two studies (21, 22), the described technology was in the acceptance (4) phase. Three (23–25) studies do not describe a specific technology application. In two (26, 27) studies the exact level of the applied technology could not be assigned to one level only.

Looking at technologies applied as described in other sources, most (*N* = 26 out of 32) were in the envisioned (1), operational (2), and/or the applied (3) phase. Six technologies were (almost) in the acceptance phase. These technologies contribute to keeping a daily rhythm (28–30) or contribute to calming down a person with dementia (31) or contribute to physical activity (32) or safety/comfort (33).

The described technology applications (literature studies and other sources) mainly focused on lowering the care burden (*N* = 32). In some (*N* = 18) cases, the described technologies may contribute to for example lower the care burden and improve sleep. In Table 1 the results are shown, the technology applications may contribute to e.g., improve sleep, but this is not necessarily scientifically proven in the included studies.

**Table 1:** Overview of the technological applications contributing to lower the care burden, improve sleep, and/or reducing social isolation.

	**Scientific**	**Other**
**Contribute to**	***N*=**	***N*=**
Lower care burden	13 (21, 23, 24, 34–43)	19 (28–31, 33, 44–57)
Improve sleep	5 (13, 43, 58–60)	6 (61–66)
Social isolation	0	4 (67–70)
Social isolation/Improve sleep	1 (71)	0
Lower care burden/Improve Sleep	14 (22, 25–27, 72–81)	2 (32, 82)
Lower care burden and social isolation	0	1 (83)

In Tables 2–10, the technology applications both found in the literature studies and other sources are ordered according to the level of the Pyramid of Technology. The literature studies are ordered on publication year as well and we add the kind of study. We take into account the Pyramid of Evidence when it comes to the level of evidence, in which Systematic Reviews of RCT are the highest level and Opinions the lowest (85).

**Table 2:** Technology applied in literature studies.

**References**	**Pyramid of technology level**	**Technology**	**Context**	**Study**	**Duration**	**Number of participants**
Tsolaki et al. (34)	1	Not-intrusive sensors, ambient depth camera, tags, wristwatch, and voice records. Monitoring with technology is only beneficial when there follows an action when necessary.	PwD living alone	O	N/A	N/A
Cahill et al. (35)	2	Automatic Night and Day calendar, lost item locator, automatic night lamp, gas cooker device, and picture button telephone	PwD alone or together	C	N/A	20
Kang et al. (36)	2	Wearable and environmental technology for monitoring and alerting. Also an electronic pillbox and sensors and monitors.	N/A	R	N/A	N/A
McKenzie et al. (37)	2	Safe Home Program, for ongoing surveillance, provision of care, prevention of injuries, and improving home safety. Using motion sensors, camera, proximity range alarm, medication alarm, locating technology, multiple sensors for safety, and detectors (e.g., smoke and water).	PwD alone or together	R/D	3 m	60
Meiland et al. (38)	2	Rosetta system, a mobile device, sensors, automatic detection of emergencies. System offers reminders for activities, a picture dialing system, radio and music button, activity support [e.g., making coffee and safety warning (e.g., an open window), monitoring, prevention and emergency response, fully automatic detection of emergencies].	PwD alone or together	P	N/A	50
Gitlin et al. (39)	2	WeCareAdvisor, a web-based platform that provides information about dementia, tips and an approach to create treatment plans (based on behaviors) with tips and evaluation. The system is installed on an iPad.	PwD alone or together	RCT	1 m	57
Lazarou et al. (40)	3	Wearable sensors to detect sleep patterns, physical activity, and activities of daily living	PwD alone	C	3/4 m	4
Husebo et al. (42)	3	Seven studies used wearable technologies (multiple sensor systems, ankle or wristband, or a combination of both). They identified 12 studies that used sensor-based motion detection (non-wearable). Twelve studies were found that utilized sensors rays placed in the living environment. Three additional studies were added with a unique technological approach, a robot, a tablet tool for text analysis.	N/A	SR	N/A	N/A
Rawtaer et al. (41)	3	System to detect changes in behaviors with passive infrared motion sensors, beacon tags, medication box with sensor, bed sensor, and a wearable.	PwD alone	CS	2 m	49
Leyhe et al. (84)	3	Sensor system to support age in place, with wearable sensors, ambient sensors, or a combination of these. Sensors which can monitor and share activities.	N/A	O	N/A	N/A
Topo et al. (21)	4	Automatic Night and Day calendar	PwD alone or together	A	3 m	50
Abraha et al. (23)	N/A	Sensory stimulation interventions, cognitive/emotion-oriented interventions, behavior management, and other therapies (e.g., exercise therapy, animal-assisted therapy). Music therapy and behavioral management therapies were effective for reducing BPSD.	N/A	SR	N/A	N/A
Anderson et al. (24)	N/A	Several interventions are discussed. Behavioral interventions with the COACH system. A cognitive assistive technology that provides task guidance (e.g., hand washing). BESI is a system of body-worn and in-home sensors to detect agitation and its environmental triggers.	Home and nursing home	R	N/A	N/A

*1, envisioned; 2, operational; 3, applied; 4, accepted; C, Case study; A, Assessment study; R, Review; P, Participatory design study; D, Demonstration project; O, Opinion; SR, Systematic Review; CS, Cross-sectional study; m, months; N/A, not applicable*.

**Table 3:** Technology applied in studies found from other sources.

**Name**	**Pyramid of technology**	**Technology**
SEM (Watchsem)	1	SEM (Sleep, Eat, Move) is a watch application to support people with early-onset dementia. The application can support daily rhythm based on sleeping, eating, and moving. With reminders, by using recognizable pictures and voice the app supports to keep daily rhythm (44).
Empathic Dwelling	2	The Empathic Dwelling is a research program that focuses on three main building elements in a building, floor, walls, and roof. The dwelling thinks along with the person with dementia, by using smart sensors for example. COOK3R is a smart and interactive cooktop that thinks along with a person with dementia during cooking. The COOK3R gives spoken instructions, light and sound signals, and automatically stops the heating when the food is ready. Interactive Living is using projection, light and sound signals to support people with dementia with their daily activities. For example, getting up on time, eating and drinking, and going to bed on time (45).
HAGU (Jingcailiu)	2	Hagu is a vest that tightly enfolds its owner giving the sensation of being hugged, this can be supportive in making life more comfortable (46).
Ritme	2	Ritme can support the daily structure of someone. The app generates a signal at a certain time, and this only stops when someone has scanned the correct QR code (for example, in the kitchen) (47).
AAL eWare	2	AAL project “Early Warning Accompanies Robotics Excellence” is focused on improving the lifestyle of people with dementia and their caregiver (s). In the project, lifestyle monitoring is integrated with social robotics (48).
AAL ReMIND	2	ReMIND is an AAL project in which a nursing robot (James) is combined with a tablet. It should be an interactive agenda, library of music, pictures, and exercises that guarantee the desired stimulation of patient and caregiver (49).
AAL CARE smart sensor	2	The AAL Care project wants to realize an intelligent monitoring and alarming system. (50)
AAL MedGUIDE	2	AAL project MedGUIDE developed a digital platform that brings together informal caregivers, care professionals, pharmacists, and people with dementia themselves. The system collects subjective (self-report) and objective data (sensors), to provide an up-to-date view on the state of needs of people with dementia. MedGUIDE uses big data analysis to detect changes in patients' routines, to minimize the side effects of medication. The system supports medication adherence by direct reminders (51).
AAL MEMENTO	2	AAL MEMENTO is a tool to create memories in everyday life. It is a sort of picture, voice, and video diary for people with dementia (52).
TimeSteps	2	TimeSteps is an application to support people with dementia with awareness of time and remind them of appointments (53).
HUME (Mentech Innovation)	3	HUME is based on sensors, behavioral models, and machine learning and can recognize emotions (54).
Tessa (Tinybots)	3	Tessa looks like a plan pot and is capable of speaking and can provide alerts, reminders, verbal guidance, and encouragement to patients (55).
Felix (Happybots)	3	Felix is a social robot that can help people express feelings (56).
OER	3	OER is an easy-to-use music player. The music player can be used by people with dementia because of the ease of use (57).
DayClocks	4	DayClocks have different functions, it is a clock (analog, digital, day and part of the day), but family and informal caregivers are also able to send messages, appointments, and pictures to the DayClock of a person (28).
Don't forget it	4	Don't forget it is an application which helps people to remember appointments. The solution is a display with appointments and other important information (29).
Bbrain Family D2 Dementieklok	4	BBrain enables older adults to continue functioning independently longer, even with dementia. A BBrain tablet supports structure, creates tranquility and engagement. It is also possible to communicate via BBrain with messages, pictures, and video calls with other people (30).
Domotica	4	Automatic control of electronic devices in the home. The devices are connected to the internet, so you can control them remotely (33).
JustoCat	4	An interactive robotic cat, as alternative of a real pet (31).

*1, envisioned; 2, operational; 3, applied; 4, accepted*.

**Table 4:** Technology applied in literature studies.

**References**	**Pyramid of technology level**	**Technology**	**Context**	**Study**	**Duration**	**Number of participants**
Rose et al. (43)	1	Body sensors.	Home	C	5/7 w	50
Wang et al. (58)	2	Monitoring and support system, using sensors.	PwD alone	C	3 m	2
Aarts et al. (59)	2	Dynamic lighting.	Nursing home	C	N/A	6
Jones and Moyle (60)	2	Customized, removable, washable, quilted cover placed over a pillow. With music and Intrasound TechnologyTM.	Care facility	F	4 w	4
Pu et al. (13)	3	Paro robot seal.	Nursing home	RCT	6 w	41

*1, envisioned; 2, operational; 3, applied; F, Feasibility study; C, Case study; RCT, Randomized Controlled Trial; m, months; w, weeks; N/A, not applicable*.

**Table 5:** Technology applied in studies found from other sources.

**Name**	**Pyramid of technology**	**Technology**
Muziek in de nacht/Music at night	2	“Muziek in de nacht” is an application for people with sleep issues. The app responds to voice sounds, to then calm the client with quiet music or a familiar voice, which is played automatically (61).
Sparckel	2	Sparckel is a biodynamic lighting armature. In this lighting the illuminance level and the color temperature are combined in the right proportion and varied throughout the day, resembling a daylight curve (62).
Brise AI Guardian Angel	2	BRISE AI Guardian Angel senses and analyses your home environment, it diagnoses and recommends action to protect you and intelligently improves your home or work ecosystem to help prevent, ease, solve asthma and allergic symptoms for a healthy deep breath (63). An uncomfortable environment, can trigger BPSD.
Somnox	3	Somnox is a robot-like cushion that helps people fall asleep, by calming the mind and body (64).
Lyla Sleep Coach	3	Lyla Sleep Coach is based on cognitive behavioral therapy. Users of the application learn to get rid of their incorrect sleep behavior and adopt the correct behavior 6 weeks (65).
Qwiek.snooze	3	Qwiek.snooze is a smart music pillow designed for people with dementia to have a better sleep. The pillow helps to relax and to fall asleep with music (66).

*2, operational; 3, applied*.

**Table 6:** Technology applied in studies found from other sources.

**Name**	**Pyramid of technology**	**Technology**
Tooloba	1	Tolooba develops software that retrieves ‘hidden' memories of people with dementia. With the software, memories activate by using images selected by an algorithm, in this way the software can help to reconnect, improve happiness, wellbeing, and interaction between a person with dementia and carer(s) (67).
AAL Sense-Garden	2	The Sense-Garden is a room in which people with dementia accompanied by a caregiver or family member walk into his/her history and memories (68).
AAL MI-Tale	2	An interactive game to recall memories of people with dementia. Using historical pictures and videos, also from the user (69).
123 Familie	3	123 Familie is a video call application for people with dementia. The application has an intuitive interface and is easy to use. It is also possible to use an automatic pick-up function when necessary (70).

*1, envisioned; 2, operational; 3, applied*.

**Table 7:** Technology applied in literature studies.

**References**	**Pyramid of technology level**	**Technology**	**Context**	**Study**	**Duration**	**Number of participants**
Tanaka et al. (71)	2	Human-type communication robot.	PwD alone	RCT	8 w	34

*2, operational; RCT, Randomized Controlled Trial; w, weeks; N/A, not applicable*.

**Table 8:** Technology applied in literature studies.

**References**	**Pyramid of technology**	**Technology**	**Context**	**Study**	**Duration**	**Number of participants**
Rowe et al. (72)	2	CareWare gives alerts when a person leaves their bed and tracks them as he/she moves about the house.	PwD alone or together	RP	12 m	53
Rowe et al. (73)	2	Monitoring system which gives alerts when a person leaves their bed.	PwD alone or together	CT	1 y	49
Martin et al. (74)	2	NOCTURAL, see Agusto et al. (75).	Home	P	3 m	8
Augusto et al. (75)	2	NOCTURAL, the system contains audio activity (music and spoken word), visual activity (images displayed on a device), combination (audio and visual), sequenced lighting guidance.	PwD alone or together	UCD	N/A	9
Radziszewski et al. (76)	2	Night Assistance System (NAS) with a monitoring and assistance phase. Collects environmental data with sensors and physiological data from worn sensors. Effectors are e.g., a table lamp, LED bulbs, light reminders and paths, and a media center.	PwD alone or together	E	3 m	4
Williams et al. (77)	2	FamTechCare intervention provides dementia strategies based on video recordings that are assessed by experts.	PwD together	RCT	3 m	42
Ault et al. (78)	2	NWDD (Night-time Wandering Detection and Diversion system), an assembly of components from Samsung Smart Things, SONOS, Ideal Security, and Ecolink sensors. There is a motion sensor, a multipurpose sensor, a door and window sensor, and a pressure mat (in bed). The system reacts when the person with dementia leaves the bed.	PwD alone or together	PL	12 w	5
Obayashi et al. (79)	2	Combination of technology, a sheet-shaped body vibrometer with a communication robot.	Nursing home	PC	4 w	15
Lussier et al. (80)	2	AAL-system with passive infrared sensors, magnetic contact sensors, and smart electric switches. The system can identify trends in sleep, outing, cooking, mobility and hygiene activities.	PwD alone	C	490 d	1
Gong et al. (26)	2 (drybuddy)	A system, with a sensing layer with mainly three types of sensors for detecting wetness, nighttime agitation, and speech outbursts. In the system, the DryBuddy device, microphone, and TEMPO sensors are used.		C	5/7 d	12
	3 (TEMPO)		PwD alone or together			
	4 (microphone)					
Saragih et al. (27)	2 (Kabochan) 2 (Bomy) 3 (NAO) 3 (Paro)	In 12 of the studies Paro is used (one combined with NAO robot), in 2 studies social robot Kabochan is used and in one study, robot Bomy is used.	Nursing home and home	SR	N/A	N/A
Moyle et al. (81)	3	PARO-robot seal, a therapeutic pet-type robotic. It is used as a promising alternative to animal-assisted therapies for residents with dementia. Paro can move his fins and make sounds.	Nursing home	CRCT	10 w	28
Jones et al. (22)	4	Personal music on a MP3-player.	N/A	SR	N/A	N/A
Van Hoof et al. (25)	N/A	Light can improve cognition, mood, and behavior, sleep, and vision when properly installed in the dwelling of a person with dementia	PwD alone or together	O	N/A	N/A

*2, operational; 3, applied; 4, accepted-E, Evaluation; PC, Proof-of-concept; CRTC, Clustered Randomized Controlled Trial; UCD, User Centered Design; P, Participatory design study; RP, Randomized Pilot; CT, Controlled Trial; PL, Pilot; O, Opinion; SR, Systematic Review; C, Case study; d, days; w, weeks; m, months; y, year; N/A, not applicable*.

**Table 9:** Technology applied in studies found from other sources.

**Name**	**Pyramid of technology**	**Technology**
VitaPillow	2	Vita is an interactive cushion for people with dementia. The cushion can play music or personal audio files by touching one of the six textile touchpads (82).
Silverfit Alois	4	SilverFit contains activities to stimulate people to move, make contact, do a cognitive game, or relax. Silverfit is a system with a display and a 3D motion-sensing camera to detect the player(s) (32).

*2, operational; 4, accepted*.

**Table 10:** Technology applied in studies found from other sources.

**Name**	**Pyramid of technology**	**Technology**
CareRiing	3	CareRiing can be seen as a smart answering machine, this is filled with phrases and words by family. A person with dementia can call the Care-Riing number at any time of the day and with speech recognition and AI the correct phrases will be played (83).

*3, applied*.

### Lower Care Burden

As mentioned (Table 1), 13 studies describe technology applications that contribute to lower the care burden of an informal caregiver. Besides that, 19 technology applications are identified from other sources.

Most (*N* = 32) of the technology applications contribute to lower the care burden of the informal caregiver of a person with dementia. There are different types of technology applications found and also the phases according to the Pyramid of Technology differ. The Automatic Day and Night calendar were described twice in literature (21, 35), and the same sort of technology (28, 29, 53) was found in other sources. This sort of technology works with reminders to support daily rhythm. Concerning the use of in-home sensors, nine studies (24, 34, 36–38, 40–42, 84) described the use of in-home sensors, and four applications (33, 48, 50, 51) from other sources utilize them as well. Wearable devices are mentioned six times (24, 36, 40–42, 84) in the included studies and six times (31, 44, 46, 47, 53, 54) in other sources. In the scientific papers, no robot-based technology application was found, four applications (31, 48, 55, 56) from other sources make use of robotics.

### Improve Sleep

Five studies indicated a technology application that contributes to improved sleep and six applications were added from other sources.

To improve sleep, 11 technology applications were detected. None of these applications were yet in the accepted phase according to the Pyramid of Technology. Regarding scientific evidence, one study (58) was published in 2010, the others (*N* = 4) 2015 or later. Furthermore, three were case studies (58, 59, 86), one a feasibility study (60), and one RCT (13). Looking at the applications, two (59, 62) technology applications were based on lighting, and three were a sort of pillow people can use. Music was used in four technology applications (60, 61, 64, 66) and sensor technology was used in two applications (58, 63).

### Social Isolation

Four technology applications targeting the reduction of social isolation were only found in other sources, none in scientific literature.

Three (out of 4) technology applications (67–69) focused on memories of people with dementia to support the conversation. One technology application (70) was used for video calls.

### Social Isolation and Improve Sleep

In this review, one technology application was found which targets the reduction of social isolation and improving sleep.

The technology application was described in a scientific paper presenting the results of an RCT using a human-type communication robot (71).

### Lower Care Burden and Improve Sleep

This paragraph describes the technology applications aiming at lowering the care burden and improving sleep. A total of 16 technology applications are given in Tables 8, 9 below.

The technology applications intending to lower care burden and improve sleep were located relatively low in the Pyramid of Technology, only two (32, 87) were in the accepted phases. The others (*N* = 13) were in the operational and/or applied phase, from one application this information was missing24 (25). One technology application was described twice in literature (74, 75). Also, Paro, the robot seal is mentioned twice (27, 81) in literature. Six technology applications were a combination of sensors and another technology (72–75, 78, 79).

### Lower Care Burden and Social Isolation

One technology application from other sources, the CareRiing innovation, targeted to lower care burden and reduce social isolation.

## Discussion

The hypothesis was that technology applications contribute to (a) lower the care burden, (b) improve sleep, and therefore (c) may contribute to reducing social isolation of the informal caregivers of people with dementia. Therefore, the research question for this exploration was “Which technology is available to caregivers to reduce the negative effects of nightly activities from the person with dementia in the home setting?.”

Social isolation of informal caregivers of people with dementia can have different causes such as stress, shrunken personal space, and diminished social interaction caused by the care role, feelings of powerlessness, and helplessness (88). As dementia progresses, the caregiver has to give up their job and/or has no time for any social activities or gatherings. Their lives only revolve around the care of the person with dementia. Informal caregivers may be feeling ignored, abandoned and isolated (89). According to Pearlin et al. (90) stress of an informal caregiver is caused by several contextual factors: the primary stressors of the illness (such as BPSD), secondary role strains, and intrapsychic strain such as personality, competence, and role captivity of the informal caregiver. Caregiver burden has been defined as a negative reaction to the impact of providing care on the informal caregiver's social, occupational, and personal roles by Sherwood et al. (91). Caregiver burden can increase by the deterioration of dementia, as caregivers find that they must supervise a person more closely. Besides that, the person's cognition may change unpredictably during the day (from lucidity to confusion), making social relationships precarious (91).

In this review, we identified technology applications that contribute to lower the care burden of an informal caregiver of a person with dementia. These technologies for example use reminders to keep a daily rhythm or can give alerts and/or information due to a monitoring system that uses sensors. Furthermore, we also identified technologies (*N* = 16) that contribute to a lower care burden but also to improve sleep. These technologies are for example technologies that can be used to calm a person with dementia or can support a caregiver and/or person with dementia during the night with alerts or music/lights (see Table 8). Sensor-based technology such as waring systems as well as robot technology such as Tessa and Paro were found as well. One technology application is designed to lower the care burden and to have a positive effect on the reduction of social isolation of the person with dementia (in this case).

In regard to “improve sleep,” robot-, sensor-, music- and lighting-based technologies were included. It is known that enough light during the day can improve mood and behavior, cognition, vision, and sleep (25, 92). These technologies can indirectly support sleep of persons with and/or without dementia. The robot included is regarding improved sleep is the seal Paro which is also included in lowering the care burden. (Music) Pillows can support people with dementia to calm down and fall asleep and sleep through.

Sleep is not always mentioned or measured specifically in the literature studies. Because sleep could be defined in the literature as part of the activities of daily living. So, for further research, it is important to define the daily activities and on which activities technology applications will contribute. In addition, it could also be mentioned as a side effect of other interventions. In other studies, improved sleep can be a side effect of other interventions.

This review had a focus on lowering care burden, improving sleep and reducing social isolation. In our opinion, a lower care burden and a higher sleep quality can contribute to reducing social isolation. However, for this review, we did not search on technology applications specifically focused on social contact even though that kind of technology is available. Khosravi et al. (93) identified eight different technologies that have been applied to reduce social isolation, namely general ICT, video game, robotics, personal reminder information and social management system, asynchronous peer support chatroom, social network sites, telecare, and 3D virtual environment. However, the study was not focused on dementia care in home settings. To our knowledge, limited studies describe the use of technology applications that have a focus on care burden, lack of sleep, and social isolation of informal caregivers of people with dementia. Goodman-Casanova et al. (94) described a support, monitoring and reminder system and a television-based assistive service to use during the COVID-19 pandemic. Memory café online described by Masoud et al. (95) also arose due to COVID-19. A telehealth solution is mentioned by Kabir et al. (96), this solution is an application on a smartphone or tablet. With this application an informal caregiver gets support from peers and healthcare professionals. An easy-to-use telephone is described by Topo et al. (97), this kind of technology is also identified in our review.

### Limitations of the Study

In this review, we did not differentiate between studies in home-settings and elsewhere even though some of the applications currently are only available for long-term care facilities, e.g., due to pricing or complexity of the technology. This was a contentious choice, as over time, these technologies may be used in the home environment therefore it is relevant to include these technology applications. For example, we know that the social robot seal Paro is also used individually. However, no studies reported about the use of Paro in home settings although this could be valuable and relevant. Technology applications developed for bigger settings are not always appropriate to install in the home-setting and cannot always be used independently, or the investment for an individual is too high. Besides this we do not take into account the cost-effectiveness and effectiveness of the technology applications included; in further research we can look at these aspects. Technology applications which are less suitable for use at home are for example dynamic lighting, Paro, Tooloba, AAL Sense Garden, AAL ReMIND, NAO, Kabochan, Bomy and Silverfit.

The identified technology applications are categorized on lowering the care burden, improving sleep, and decreasing social isolation, by the authors based on the description of these technologies. Moreover, the assignment to a category is, done on the four-eyes principal method (20). The Pyramid of Technology (18) was used to categorize the technology applications found. Discrepancies between authors concerning the place of the application on the level of Pyramid of Technology were solved by discussion until consensus was reached. The model helped to calibrate and determine the most appropriate classification of the status of the individual technology. In that way, the model provides insight into the status of the technology and helped to reach a consensus between researchers. The second and third authors labeled technology applications used and categorized these based on abstract only to calibrate with the scoring first author. Scoring based on abstract was sometimes difficult because of missing information regarding the specific technology. In addition to this, categorizing technologies by using this level of Pyramid of Technology directs categorization in one way only. Yet, this way was chosen because it provides insight into the level of development status of the technology applications in daily practice It is used to indicate the accessibility and usability to the aging population. Another limitation is that all authors are from The Netherlands so especially the technology applications from other sources and countries are incomplete due to limitations in the accessibility of other sources outside the Netherlands. Furthermore, the search ended in May 2021, other studies after May 2021 could therefore have not been included, because some technological developments are very fast, this can be seen as a minor limitation of the study.

### Future Studies

This scoping review is part of a larger project regarding finding solutions to release the care burden of informal caregivers of people with dementia. The results of this first exploration will be discussed in one or more consultation rounds, according to Arksey and O'Mally (16) and Rumrill et al. (17). Further studies should also focus on the efficacy of the available technologies. In addition, we need to pay attention on how to achieve scaling up of technology applications for people with dementia, even beyond the use in healthcare.

## Conclusion

This scoping review identified technology applications to support caregivers of persons with dementia. Several applications were found, however, most of the applications were located at the second level of the Pyramid of Technology, namely the operational level.

A diverse group of technology applications is available to support an informal caregiver of a person with dementia, in and around the night. The technology identified supports informal caregivers in different ways. Some targeting the person with dementia, others the informal caregiver or both. Technology applications were mostly used to lower the care burden of an informal caregiver by supporting daily rhythm, by calming a person with dementia, by increasing safety in the home, or supporting communication.

## Data Availability Statement

The original contributions presented in the study are included in the article/supplementary material, further inquiries can be directed to the corresponding author.

## Author Contributions

CH determined the research and HK the method to analyze the data. CH performed data collection and preparation of the manuscript with support of HK and EH. The analysis and interpretation of the data was performed by CH, HK, and EH. All authors reviewed the results and approved the final version of the manuscript.

## Funding

This scoping review was co-funded by Regieorgaan SIA, part of the Netherlands Organisation for Scientific Research (NWO) and with PPP funding made available by Health~Holland, Top Sector Life Sciences & Health, to stimulate public-private partnerships (EXZ.EXZ.01.005). This research was co-funded by University of Applied Sciences Utrecht.

## Conflict of Interest

The authors declare that the research was conducted in the absence of any commercial or financial relationships that could be construed as a potential conflict of interest.

## Publisher's Note

All claims expressed in this article are solely those of the authors and do not necessarily represent those of their affiliated organizations, or those of the publisher, the editors and the reviewers. Any product that may be evaluated in this article, or claim that may be made by its manufacturer, is not guaranteed or endorsed by the publisher.
